# Site-Specific, Covalent Immobilization of Dehalogenase ST2570 Catalyzed by Formylglycine-Generating Enzymes and Its Application in Batch and Semi-Continuous Flow Reactors

**DOI:** 10.3390/molecules21070895

**Published:** 2016-07-11

**Authors:** Hui Jian, Yingwu Wang, Yan Bai, Rong Li, Renjun Gao

**Affiliations:** Key Laboratory for Molecular Enzymology and Engineering, the Ministry of Education, School of Life Science, Jilin University, Changchun 130012, China; jianhui18686@163.com (H.J.); by0330@126.com (Y.B.); ryong_li@163.com (R.L.)

**Keywords:** immobilization, dehalogenase, formylglycine-generating enzyme, *Sulfolobus tokodaii*

## Abstract

Formylglycine-generating enzymes can selectively recognize and oxidize cysteine residues within the sulfatase sub motif at the terminus of proteins to form aldehyde-bearing formylglycine (FGly) residues, and are normally used in protein labeling. In this study, an aldehyde tag was introduced to proteins using formylglycine-generating enzymes encoded by a reconstructed set of the pET28a plasmid system for enzyme immobilization. The haloacid dehalogenase ST2570 from *Sulfolobus tokodaii* was used as a model enzyme. The C-terminal aldehyde-tagged ST2570 (ST2570CQ) exhibited significant enzymological properties, such as new free aldehyde groups, a high level of protein expression and improved enzyme activity. SBA-15 has widely been used as an immobilization support for its large surface and excellent thermal and chemical stability. It was functionalized with amino groups by aminopropyltriethoxysilane. The C-terminal aldehyde-tagged ST2570 was immobilized to SBA-15 by covalent binding. The site-specific immobilization of ST2570 avoided the chemical denaturation that occurs in general covalent immobilization and resulted in better fastening compared to physical adsorption. The site-specific immobilized ST2570 showed 3-fold higher thermal stability, 1.2-fold higher catalytic ability and improved operational stability than free ST2570. The site-specific immobilized ST2570 retained 60% of its original activity after seven cycles of batch operation, and it was superior to the ST2570 immobilized to SBA-15 by physical adsorption, which loses 40% of its original activity when used for the second time. It is remarkable that the site-specific immobilized ST2570 still retained 100% of its original activity after 10 cycles of reuse in the semi-continuous flow reactor. Overall, these results provide support for the industrial-scale production and application of site-specific, covalently immobilized ST2570.

## 1. Introduction

Enzymes are biocatalysts that catalyze reactions under mild conditions with high efficiency, and stereospecificity or enantioselectivity, and they also reduce or avoid adverse conditions. Enzymes are used in organic synthesis, brewing, food, medicine and other fields. However, enzymes exhibit limited mechanical and storage stability for some reactions and cannot be reused. With the emergence of protein modification and immobilization techniques, enzymes have become conducive to automatisation and continuous use for industrial production.

Enzyme immobilization may alter enzyme property and performance in many different ways, which depends on the methods used, a proper immobilization may improve stability and, in certain cases, activity, while a bad selection of the immobilization strategy may drive to the enzyme inactivation and even decrease enzyme stability [1]. Physical methods including physical adsorption and entrapment etc., which are simple, however the immobilized enzymes with those methods maybe leaked out from the matrix during operation [2]. The immobilization of enzymes via multi-point chemical covalent binding sometimes may result in a certain loss of observed activity, however, the chemical stability will be significantly enhanced and the enzyme leakage could be minimized [3]. In addition, immobilization may provide other benefits such as creating compatible environment for enzyme, and avoiding multi-layer packing of enzymes etc., such that improved specific activity may be observed after immobilization in certain cases [4]. Enzyme orientation during immobilization may play a critical role in the recovered activity, for example, when the substrate is large and the active center is oriented towards the surface of the support this may raise some steric hindrances, or when the interaction of a specific area of the enzyme with the enzyme may drive to their inactivation [5]. Modifying enzymes by adding a tag can maintain enzyme activity and avoid enzyme inactivation caused by chemical reactions. Various techniques have been proposed for the chemical modification of proteins [6]. Introduction of short peptides to the target protein is a commonly useful method to achieve selective and site-specific modification of a proteins. With this method, target proteins can be recognized selectively and modified by a co-expressed enzyme in vivo or in vitro [7,8,9,10,11,12,13]. The reaction between the protein and the supports is therefore more controlled. Table 1 lists the enzyme-mediated systems used for the modification of target proteins. Prokaryotic formylglycine-generating enzyme (FGE) is used for protein labeling and was first identified in 2003 [14]. Several researchers reported that FGE can recognize a six-residue sulfatase submotif (LCTPSR) and oxidize the cysteine residue selectively within the motif to form an aldehyde-bearing formylglycine (FGly) residue [15,16]. The target protein can be fused with a LCTPSR submotif for modification by a co-expressed FGE. Thus, the free aldehyde group on the surface of the target protein can be used for site-specific covalent binding to amino-functionalised fluoresceins or immobilization supports through a Schiff base reaction [10].

Haloacid dehalogenases exhibit a potential for pharmaceutical and chemical industrial use and for the remediation of contaminated land [17]. In previous work, haloacid dehalogenase from the thermophile archaeon *Sulfolobus tokodaii str.7* (ST2570) was cloned and characterized [18]. This enzyme belongs to the L-2-halogen acid (L-2-HAD) superfamily and is vital for industrial applications because of its efficient catalytic activity, stereoselectivity, thermal stability and tolerance to a wide pH range. As a monomer globin, ST2570 was a suitable model enzyme for testing site-specific covalent immobilization [19].

**Scheme 1:**
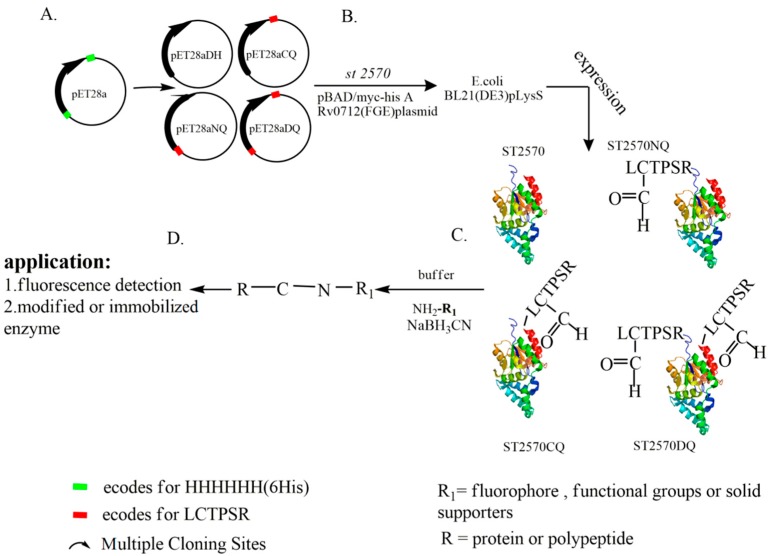
Schematic of the *E. coli* co-expression system used for the aldehyde tagging of proteins in vivo. (**A**) the reconstructed plasmids; (**B**) the expression of four kinds ST2570 that are either wildtype enzymes or modified enzymes with aldehyde groups in different positions; (**C**) the one-step specific immobilization of ST2570; (**D**) the immobilized ST2570.

Many scientists have successfully fused a sulfatase sub-motif (also called an “aldehyde tag”) to target proteins by PCR reaction using a specific primer design before the construction of an expression plasmid. In this study, the pET28a plasmid was reconstructed, as shown in Scheme 1A. The gene encoding the peptide LCTPSR was introduced at different positions in pET28a, and a new set of vectors were obtained: pET28aDH, pET28aCQ, pET28aNQ and pET28aDQ. Thus, the sulfatase submotif can be easily fused to any target protein at different positions without designing specific primers. Two expression plasmids containing the ST2570 gene and FGE gene would be transformed into bacteria for co-expression. The host that retains both plasmids will co-express ST2570 and FGE in the presence of L-pectinose and isopropyl β-d-1-thiogalactopyranoside (IPTG). In this study, four proteins that are either wildtype enzymes or modified enzymes with aldehyde groups in different positions will be obtained (Scheme 1B).

It is well-known that both the method and the support used might impact the efficiency of enzyme immobilization. Inorganic supports can form manageable particles with large surface areas, are reactive towards derivatizing/functionalizing agents and present excellent thermal and microbial resistance, reasonable mechanical strength and chemical resistance under operational conditions. On the other hand, mesoporous supports not only exhibit a very high specific surface (up to 500 m^2^/g), but can also host the enzyme molecules within pores and protect against physical and mechanical damages [20]. Fortunately, SBA-15 has all the advantages above and was selected as the support. Amino functionalized SBA-15 is also low-cost and convenient for the one-step specific immobilization of ST2570 (Scheme 1C).

## 2. Results and Discussion

### 2.1. Expression and Purification

**Figure 1:**
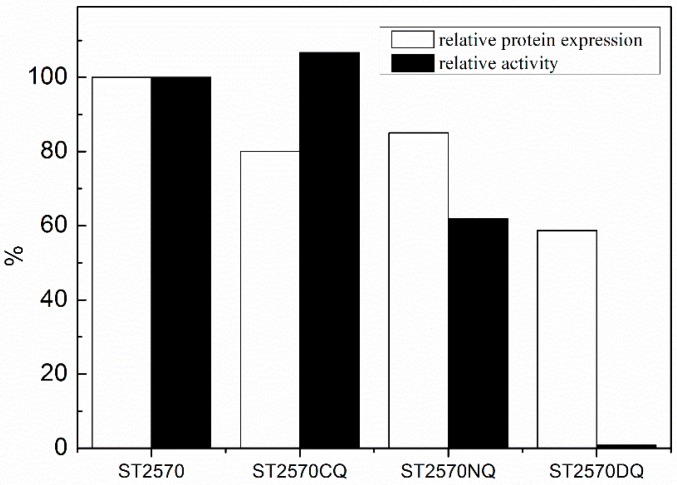
Effect of the fused aldehyde tag on the expression level and activity of the recombinant protein ST2570.

The aldehyde tag (LCTPSR) was ligated into the C or N-terminal or both terminals of ST2570 using the reconstructed plasmid pET28a. However, results showed that the aldehyde tag variably affected the protein expression level and enzyme activity (Figure 1). Unlike wildtype proteins, all of the aldehyde-tagged ST2570 reduced the level of expression, which can evidently affect the enzyme activity. The C-terminal tag improved the activity of enzyme, despite a reduced level of expression, but the enzyme with double-sided aldehyde tags lost almost all activity. These results indicated that the fused polypeptide tag affects protein folding and the tertiary structure.

### 2.2. Labeling of Recombinant Proteins with Fluorescence Probe

**Figure 2:**
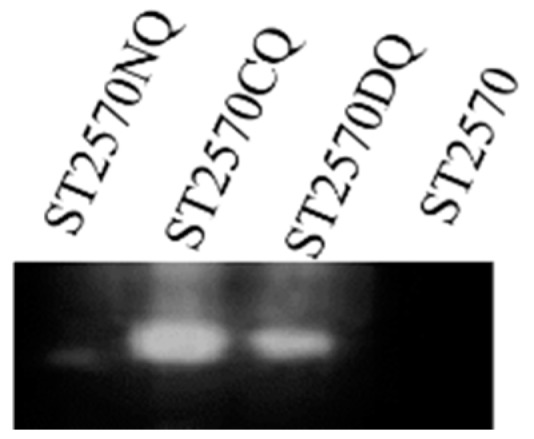
Selective labelling of aldehyde tagged proteins with Alexa Fluor 647 C5-aminooxyacetamide.

Although many protein modification methods were developed, these methods usually resulted in the selective labeling of a given functional group rather than the site-specific labeling of one residue in a given protein [8]. Thus, the fused aldehydes were used for site-specific protein modification and identification with an aminooxy functionalised probe. A panel of aldehyde-tagged proteins was incubated with Alexa Fluor 647 C5-aminooxyacetamide and the reaction was analyzed by SDS-PAGE and Odyssey Near Infrared Laser Imaging system (Figure 2). In contrast, the wildtype protein without an aldehyde tag demonstrated no signals, the C-terminal modified proteins showed strong signals. Additionally, the N-terminal and double-terminal modified proteins exhibited weaker signals. Some LCTPSR peptide tags in these proteins may be covered during the process of protein folding, and could not be modified by the co-expressed FGE. We selected ST2570CQ for immobilization because of its excellent activity and the availability of free aldehyde groups.

### 2.3. Modification of SBA15

Silica-based mesoporous materials have been used as enzyme supports, biomarkers and biosensors in medicine. However, its direct use as a support for enzyme immobilization is limited because of its inert nature. Thus, chemically modifying the surface of these materials is necessary to introduce an active group, and the most common modifier is aminopropyltriethoxysilane (APTES, C_9_H_23_NO_3_Si) [21,22,23]. As shown in Table 2, SBA-15 exhibited improved loading capacity, and the enzyme showed better immobilization efficiency (IE) but worse retention of activity (R) when immobilization was performed using support with either deficient or excessive modification. This outcome could be due to the random combination and aggregation of enzymes through physical absorption, which can enhance the steric hindrance effect and prevent the formation of the monolayer. Moreover, over-modification with APTES will result in increased steric hindrance because of the high protein density. Therefore, SBA-15 modified with 2% APTES (*v*/*v*) was selected for the next experiment.

### 2.4. Immobilization of ST2570CQ

One-step immobilization and purification of ST2570CQ were simultaneously performed through a Schiff base reaction between the amino groups in the support and the free aldehyde groups in the enzyme. This approach avoids further purification of proteins before the immobilization. The amount of enzymes bound to the carrier and the activity of the immobilized enzyme depend on conditions during immobilization, such as the availability of linking groups, pH, temperature, coupling time and initial enzyme amount. To achieve the optimal immobilization efficiency (IE), retention of activity (R) and immobilized enzyme activity, the conditions for immobilization were optimized.

**Figure 3:**
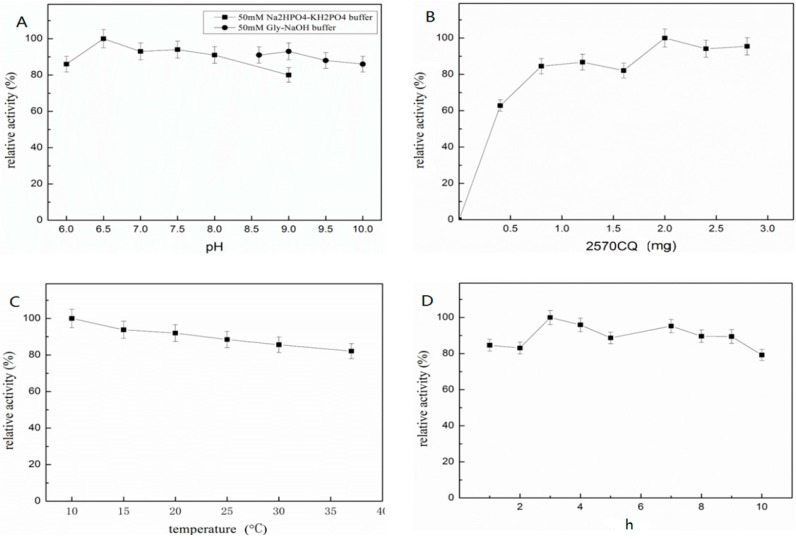
Conditions for the covalent immobilization of ST2570CQ on SBA-15. (**A**) The optimization of pH for the immobilization system using either, 50 mM PB buffer (pH 6.0–9.0) or 50 mM Gly-NaOH buffer (pH 8.6–10.0); (**B**) The optimization of the initial amount of enzyme per 10 mg support for immobilization system; (**C**) The optimization of temperature for immobilization system; (**D**) The optimization of time for the immobilization. The immobilization system consisted of 2 mL 50 mM PB buffer (pH 6.0) containing 10 mg support in a 20 mL reactor. We set the best enzyme activity as 100%. Three trials were performed for each group.

The ionization state of enzyme molecules was affected by the pH of the buffer used in the immobilization process. Figure 3A shows the effect of pH on the activity of the enzymes during immobilization. The relative activity of immobilized ST2570CQ on SBA15-NH_2_ did not vary from pH 6.0 to 10.0. The highest activity for immobilized enzymes, was observed at pH 6.5. The relative activity of the immobilized enzyme significantly increased when the initial crude ST2570CQ amounts used in the immobilization were changed from 0 to 3.0 mg per 10 mg of the carrier. However, the enzyme reached steady-state when its amounts was greater than 2.0 mg (Figure 3B). The operating temperature of immobilization was 10–37 °C, and the immobilization time ranged from 1 h to 10 h (Figure 3C,D). The optimal temperature for immobilization was 10 °C for more than 3 h. The optimal conditions for immobilization are 2 mg of crude enzyme per 10 mg of support in 50 mM PB buffer (pH 6.5) and incubation for 3 h at 10 °C. Under the optimal conditions of immobilization, the enzyme loading capacity can be as much as 108 mg/g support, and the immobilized enzyme activity can be as much as 450 U/g immobilized enzyme.

### 2.5. Characterization of Immobilized ST2570CQ

#### 2.5.1. pH and Thermo-Stability of Immobilized ST2570CQ

The pH and thermo-stability of an enzyme are important parameters for exploring potential industrial applications. ST2570 is a thermophilic protein that is stable at 70 °C. In this part of the study, the thermal stability was investigated by incubating either the free or immobilized enzyme at 90 °C for a period of time. The remaining activity was monitored after each incubation time. All the immobilized enzymes used were prepared with the optimal conditions described at the end of Section 2.4.

**Figure 4:**
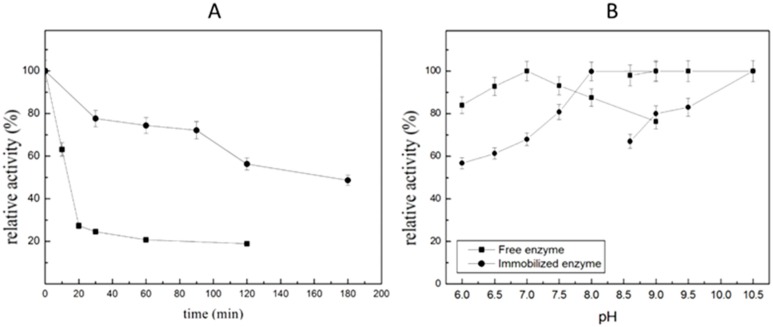
The thermal and pH stability of free and immobilized ST2570CQ: (**A**) The thermal stability of free and site-specific immobilized ST2570CQ; (**B**) The pH stability of free and immobilized ST2570CQ. (**■**) free enzyme (●) immobilized enzyme; 50 mM PB buffer (pH 6.0–9.0) and 50 mM Gly-NaOH buffer (pH 8.6–10.5). All the immobilized enzymes used were prepared with the optimal conditions described at the end of Section 2.4. The conditions for the activity assay were constant stirring at 110 rpm for 5 min in 70 °C water-bathing constant temperature vibrator. Three trials were performed for each test.

As shown in Figure 4A, when immobilized ST2570CQ was incubated for 2 h, it still retained 60% of its activity, which was still three times higher than that of free enzyme. The stability of various immobilized enzymes can be frequently improved by inorganic supports because inorganic supports have greater dimensional stabilities [24]. Inorganic supports always are capable of forming manageable particles with a high surface area, are reactive towards derivatizing/functionalizing agents and present excellent thermal and microbial resistance, reasonable mechanical strength and chemical resistance under the operational conditions [20]. The thermal stability of enzymes was speculated to drastically increase if they are attached to a complementary surface of a relatively rigid support [25,26]. The pH stability of free and immobilized enzymes is illustrated in Figure 4B. When the enzymes were incubated in buffers with different pH values for 24 h, it was found that both the free enzyme and the immobilized enzyme were stable when the pH value is higher than 7, indicating that no severe conformational change, which affects the pH stability of ST2570, occurred during immobilization [26]. As for the free enzyme that already had the best pH stability, combining it with the support did not improve the pH stability appreciably anymore.

#### 2.5.2. Operational Stabilities of Immobilized Enzyme in Batch and Semi-Continuous Reactors

**Figure 5:**
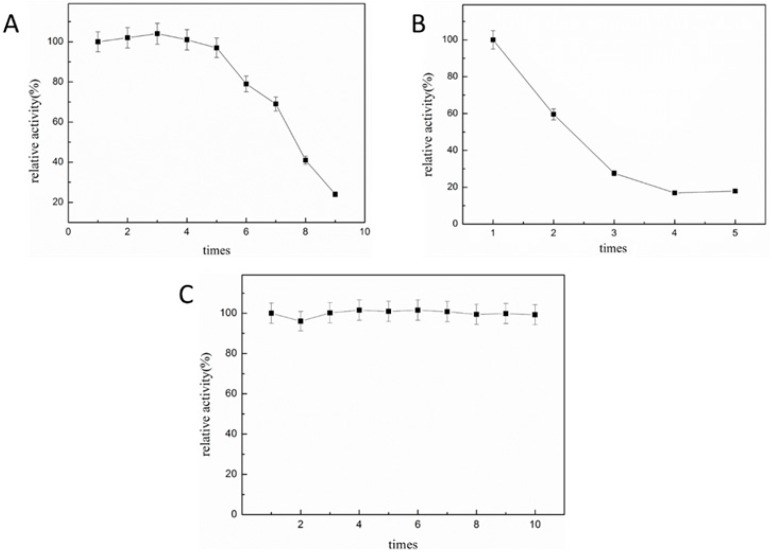
The effect of reuse on immobilized enzymes in reactors. The activity of site-specific covalently-immobilized enzymes in a batch reactor (**A**); the activity of enzyme immobilized by physical adsorption in a batch reactors (**B**); and the activity of site-specific covalently-immobilized enzymes in a semi-continuous flow reactor (**C**). The tests were carried out at room temperature.

Operational stability is the most important factor used to assess the feasibility of immobilized enzymes for industrial productions. A number of investigations were previously carried out in this area [21,27,28]. The operational stability of immobilized enzyme was determined in the batch and semi-continuous flow reactors. The results were compared in terms of activity and cycling times of the immobilized enzyme. To eliminate the influence of storage time on enzyme activity, all measurements were carried out in the same day. In the batch reaction operation (Figure 5A), the enzyme activity remained above 95% of the original activity after five cycles in the reactors loaded with 10 mg of immobilized enzyme. The result in this work shows a significant improvement in the operation stability of immobilized ST2570CQ on SBA-15 in the batch reaction compared with the immobilized enzymes in other reports [26,27,28,29,30,31]. The stability of immobilized ST2570CQ on SBA-15 by physical adsorption was also examined in the batch wise reaction (Figure 5B); which turned out the physically immobilized enzyme lost 40% of its original activity when used for the second time; and only retained less than 20% of its original activity in the fifth use; which may be ascribed to significant deprival of the enzyme from the support during the reaction agitation and recovery process. On the contrary, the activity loss of the chemical immobilized ST2570CQ on SBA-15 may be due to the deactivation of enzyme by strong shaking during washing operation. The results demonstrate that the multi-point chemical binding of ST2570CQ to SBA-15 provide a much stronger linkage and confinement for site-specific immobilized ST2570CQ than the immobilized ST2570CQ by physical adsorption [32].

In a semi-continuous flow reactor, the immobilized ST2570CQ retained almost 100% of its original activity after being used 10 times in the column (Figure 5C). The operational stability in the semi-continuous flow reactor is better than the operational stability in the batch type reactor. The difference in outcome was most likely because of the gradual loss of immobilized enzymes when the enzymes were separated by centrifugation during each round in the batch reactor. This probably did not occur in the semi-continuous flow reactor, in which the immobilized enzymes were not separated. Operational stability improved when a semi-continuous flow reactor was utilized, in contrast with the result of Bachas–Daunert [8]. Thus, the high operational stabilities obtained with immobilized ST2570CQ preparations indicate that these immobilized enzyme preparations can successfully be used for continuous production [28].

### 2.6. Catalytic Ability of Immobilized ST2570CQ

**Scheme 2:**
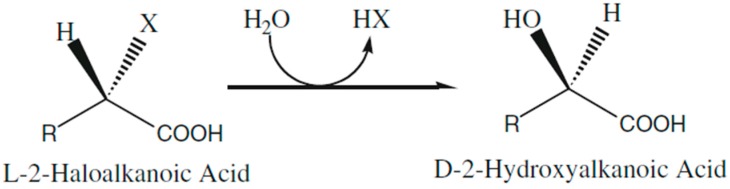
Hydrolysis reaction catalyzed by l-2-dehalogenase.

ST2570 was first reported in 2009, and it can selectively catalyze l-2-halogenated acid into d-2-hydroxy alkyl acid (Scheme 2) [18]. The immobilized and free ST2570 exhibited different catalytic abilities when l-2-haloalkanoic was used as a substrate. The highest conversion rate of l-2-haloalkanoic catalyzed by the immobilized and free ST2570 were 49.74% and 40.94%, respectively. The improvement of catalytic ability is attributed to the better thermal and operational stability of immobilized ST2570, which are the most important factors during reactions performed with high temperatures and long reaction times.

## 3. Experimental Section

### 3.1. Materials

The l-2-haloacid dehalogenase (ST2570) from thermophile archaeon *Sulfolobus tokodaiistr.7* (JCM 10545), plasmids pET28a, myc-his A Rv0712 (FGE) plasmid (add gene plasmid 16132), *Escherichia coli* BL21(DE3) pLysS (Novagen, Madison, WI, USA), mesoporous Silica Support SBA15 (550–600 m^2^·g^−1^, 7–9 nm, 0.65–0.67 cm^3^·g^−1^, Nanjing XFNANO Materials Tech Co., Ltd., Nanjing, China), (3-Aminopropyl)triethoxysilane (APTES, 98%, Aladdin, New York, NY, USA), l-2-chloropropionic acid (Alfa Aesar, Ward Hill, MA, USA), acetonitrile (HPLC Grade, Fisher Scientific, Waltham, MA, USA), ninhydrin reagent (2% (*w*/*w*) ethanol solution) and all other reagents were of analytical grade.

### 3.2. Methods

#### 3.2.1. Expression and Purification

To obtain sufficient amounts of target protein for the analysis, enzymes were produced by *E. coli* BL21 (DE3) pLysS. The method was based on a two-plasmid system in BL21 (DE3) pLysS, the first plasmid was the reconstructed pET28a with the gene encoding for the *S. tokodaii *l-2-haloacid dehalogenase (ST2570), and the other plasmid co-expressed the formylglycine-generating enzyme (FGE), which converts cysteine in the polypeptide fusion tag of LCTPSR to formylglycine (FGly) to obtain ST2570 with or without the aldehyde groups in the right place.

The aldehyde tag gene sequence was introduced to pET28a by site-directed mutation to replace the His-tag sequence. The plasmid pET28aDH was constructed by deleting the His-tag at the N-terminal of the multiple cloning site in pET28a. The plasmid pET28aCQ was constructed by replacing the His-tag at the C-terminal of the Multiple Cloning Site in pET28aDH with the gene encoding for LCTPSR. The plasmid pET28aNQ was constructed by replacing the His-tag at the N-terminal of the Multiple Cloning Site in pET28a with the sequence encoding LCTPSR. Lastly, the plasmid pET28aDQ was constructed by replacing the His-tag at the C-terminal of the Multiple Cloning Site in pET28aNQ with the sequence encoding LCTPSR.BL21(DE3) pLysS cells that carried plasmids pET28a (DH, CQ, NQ or DQ)-*st2570* and myc-his A *Rv0712* [33] were cultured in LB medium at 37 °C until an OD600 of 0.8 was obtained. The cells were induced by the addition of l-pectinose to a final concentration of 0.2% for 30 min, induced further for 16 h by 0.5 mM IPTG at 18 °C then harvested. The cell pellet was resuspended in 50 mM PB at pH 6.0, disrupted by ultrasonication in an ice bath and centrifuged at 12,000 rpm for 20 min at 4 °C. The crude extract was incubated at 80 °C for 30 min and centrifuged again. The freeze-dried protein powder was stored at −20 °C. Protein concentrations were determined using the Bradford method.

#### 3.2.2. Enzyme Assay

The 3 mL reaction system consisting of 50 mM Gly–NaOH buffer (pH 9.5) and 1 mM l-2-chloropropionic acid as a model substrate was incubated in an oscillating water bath at 70 °C for 5 min. The free or immobilized enzyme was added for further incubation in a water bath at 70 °C and shaken with a speed of 110 rpm for 5 min. The reaction was terminated by addition of 50 μL of 1 M nitric acid and 2 mL of 6% ammonium ferric sulfate solution. The system was treated with 1 mL of 0.4% mercury thiocyanate solution to produce a color reaction after removal of the immobilized enzyme. Each sample was tested three times (*p* < 0.05). The OD_460_ value for each sample was tested using a UV-1700 spectrophotometer (Shimazu, Kyoto, Japan), and the lactic acid yield combined with the chlorine ion standard curve was calculated. One unit of activity is defined as the production of 1 μmol hydrochloric acid per min at 70 °C and pH 9.5. The activity of free l-2-haloacid dehalogenase was given as U/mg_enzyme_, and immobilized enzyme activities were expressed as U/mg_enzyme_ or U/g_support_.

#### 3.2.3. Labelling of Recombinant Proteins with the Fluorescence Probe

The fluorescence-labeling reaction was performed by combining recombinant proteins and C5-aminooxyacetamide [21] and the reactions were analyzed by SDS-PAGE and Odyssey Near Infrared Laser Imaging system.

#### 3.2.4. Modification of SBA-15

SBA-15 was washed with distilled water and dried overnight at 60 °C before salinisation. Different concentrations of APTES (0%, 2%, 6%, and 10%) was added to the dried 1 g SBA-15 in 50 mL of anhydrous ethanol (*v*/*v*) and then incubated in the oscillation incubator at 80 °C and 200 rpm for 12 h. SBA-15 was washed thoroughly with anhydrous ethanol and distilled water on a filtered funnel until no APTES was detected in the filtrate, and the supports were dried overnight at 100 °C. The amount of APTES in different solutions was determined using a ninhydrin-based monitoring system described earlier by Özlem Alptekin [34]. The conditions used to initially optimize the activation of support was 2 mg of ST2570CQ per 10 mg of SBA-15-NH_2_ incubated in 50 mM PB buffer (pH 6.5) at 10 °C for more than 6 h.

#### 3.2.5. Immobilization of ST2570CQ

The amino-functionalized SBA-15 (SBA15-NH_2_) (10 mg) was incubated with 2 mL of phosphate buffer (50 mM, pH 6.0) containing 2 mg of the heat-treated ST2570 at 10 °C for 6 h of shaking at 150 rpm and reduced with 1% (*w*/*w*) NaCNBH_3_ for 1 h. Aside from the target protein, other proteins without aldehyde groups cannot establish a covalent link with SBA15-NH_2_. Thus, the immobilized enzyme was easily separated by centrifugation at 12,000 rpm for 10 min and washed twice with 1 M NaCl solution, saturated ammonium sulfate solution, and distilled water to remove the other physically adsorbed proteins. Immobilization efficiency (IE, %) and retention of activity (R, %) were calculated as follows:

(1) $$\text{IE}=\frac{\text{The activity of added enzyme}-\text{The remaining activity in the supernatat}}{\text{The activity of added enzyme}}\times 100\%$$

(2) $$R=\frac{\text{The observed activity of immobilized enzyme}}{\text{The theoretical activity of immobilized enzyme}}\times 100\%$$

The conditions for the immobilization of ST2570CQ on SBA-15-NH_2_, including pH, temperature, time and initial amount of protein per 10 mg of support, were optimized. The activity of the immobilized enzyme was determined for all the samples.

#### 3.2.6. Characterization of Immobilized ST2570CQ

In this part, the pH and thermal stabilities of the immobilized ST2570CQ were detected.

The thermal stabilities of free and immobilized enzymes were investigated by incubating both the free and immobilized enzymes at 90 °C for different lengths of time. The remaining enzyme activity was monitored after each incubation. After incubation in buffers with different pH values (pH 6.0–9.0 for 50 mM PB buffer; pH 8.6–10.5 for 50 mM Gly–NaOH buffer) for 24 h, the remaining activities of free and immobilized enzymes were detected according to Section 3.2.2.

The operational stabilities in batch and semi-continuous reactors were also investigated. For batch reactors, all reactions were carried out in a vibrating water-bathing at a constant temperature of 70 °C and shaking at 110 rpm for 5 min. The reaction mixture consists of 3 mL of 50 mM Gly–NaOH solution at pH 9.5 containing 1 mM l-2-chloropropionic and 10 mg of immobilized ST2570CQ. The enzyme was recovered by centrifugation and washed with distilled water to prepare for the next cycle. The product was then detected according to Section 3.2.2. For the semi-continuous flow reactor, 10 mg of immobilized ST2570CQ was loaded into a column (1 cm^2^ × 10 cm). The reaction mixture (mobile phase) consisting of 3 mL of 50 mM Gly-NaOH buffers (pH 9.5) and 1 mM l-2-halogenated acid was applied to a column and incubated for 10 min at 70 °C. The reaction mixture was then allowed to flow out of the column, then the product was then detected according to Section 3.2.2. The column was washed with 5 mL distilled water five times to prepare for next cycle. All the immobilized enzymes used for characterization were prepared with the optimal conditions described in Section 2.4.

#### 3.2.7. Catalytic Ability of Immobilized ST2570CQ

Rates of lactic acid production catalyzed by free and immobilized ST2570CQ were also measured using high-performance liquid chromatography (HPLC). A mixture consisting of 1 mL of 50 mM Gly-NaOH pH 9.5 containing 20 mM l-2-CPA was assigned as the control group, while 0.05 mg (9 U) and 20 mg (9 U) free and immobilized ST2570CQ were included separately in experimental groups. The conversion rate was calculated according to the following formula:

(3) $$\text{conversion rate }\left(\%\right)=\frac{\text{substrate }\left(\text{the control group}\right)-\text{the residue of substrate }\left(\text{the experiment group}\right)}{\text{substrate }\left(\text{the control group}\right)}$$

The HPLC conditions are as follows: 4.6 mm × 250 mm Waters Nova-pak C18 HPLC column; mobile phase: a mixture of 0.01 mol/L phosphate buffer (pH 2.0) and acetonitrile (8:2, *v*/*v*); flow rate: 0.6 mL/min; UV detector wavelength: 210 nm; column temperature 18 °C; volume of sample injection: 10 μL.

## 4. Conclusions

In this experiment, we prepared a panel of aldehyde tagged proteins using a pET28a plasmids system that was designed in our previous work. The C-terminal aldehyde-tagged protein (ST2570CQ) exhibited significant enzymatic properties, such as the generation of free aldehyde groups, a higher level of protein expression and improved enzyme activity. ST2570CQ was covalently immobilized to SBA-15 modified with 2% APTES ethanol solution (*v*/*v*) in the following optimal conditions: 2 mg of crude ST2570CQ per 10 mg of SBA15-NH_2_ in 50 mM PB buffer pH 6.5 and incubated at 10 °C for 3 h. The immobilized ST2570CQ exhibited a 3-fold increase in thermal stability and improved operational stability and catalytic ability than free ST2570. These improved properties increase the potential of site-specific, covalently immobilized ST2570 for industrial-scale production and application.

## Figures and Tables

**Table 1:** Enzymes-mediated systems used in protein modification.

Enzymes	Recognition Motif	Mechanism of Enzyme-Mediated System	Ref(s)
sortase A	LPXTG	A cysteine residue in sortase catalyzes the cleavage of the peptide bond between the threonine and glycine residues within the motif. Sortase will accept the N-terminus of an oligoglycine nucleophile.	[7]
microbial transglutaminase (mTG)	WALQRPH	TG2 mediates the incorporation of functionalized amines into a high-affinity Gln-substrate peptide tag.	[8]
farnesyltransferase (PFTase)	CAAX	PFTase catalyzes the transfer of a farnesyl isoprenoid group from farnesyl pyrophosphate to a sulfur atom present in the cysteine residue of the CAAX box.	[9]
formylglycine generating enzyme (FGE)	LCXPXR	FGE converts cysteine to formylglycine (FGly) within the LCTPSR motif. Proteins bearing a unique aldehyde group were chemically modified by selective reaction with hydrazide- or aminooxy-functionalized reagents.	[10]
biotin ligase (BirA)	GLNDIFEAQKIEWHE	BirA catalyzes the sequence-specific ligation of the biotin or the ketone isostere of biotin to the lysine side-chain of a 15-amino-acid acceptor peptide (AP).	[11]
microbial lipoic acid ligase (LplA)	DEVLVEIETDKAVLEVPGGEEE	LplA catalyzes the specific attachment of octanoic acid, 6-thio-octanoic acid, selenolipoic acid and alkyl azide to an engineered LplA acceptor peptide (LAP). The alkyl azide was selectively derivatized with cyclooctyne conjugates to various probes.	[12]
N-myristoyl transferase (NMT)	GXXXS	NMT specifically attaches myristate or modified myristate to peptides with amino-terminal Gly residues. Peptides with Asn, Gln, Ser, Val or Leu penultimate to the amino terminal Gly are substrates.	[13]

**Table 2:** Modification of SBA-15 with different ratios of APTES and ethanol.

Ratio of APTES and Ethanol % (*v*/*v*)	Loading Capacity mg/g_carrier_	Specific Activity U/mg	Specific Activity U/g_carrier_	Immobilization Efficiency %	Retention of Activity %
0	4.31	103	443.93	90.79	57.17
2	3.17	141.63	448.97	66.31	78.61
6	4.37	108.68	474.93	91.42	60.32
10	4.23	109.56	478.78	88.49	60.81
